# Supplementary material for: A Critical View of the Application of the APEX Software (Aqueous Photochemistry of Environmentally-Occurring Xenobiotics) to Predict Photoreaction Kinetics in Surface Freshwaters
Source: Molecules. 2019 Dec 18;25(1):9. doi: 10.3390/molecules25010009 (PMC7017383; doi:10.3390/molecules25010009)
Supplement: Supplementary file 1 [file molecules-25-00009-s001.zip › 3.2.4_gcc-4.4.0.zip!/3.2.4_gcc-4.4.0/3.2.4_gcc-4.4.0/doc/octave/html/interpreter/Creating-Permutation-Matrices.html]

Creating Permutation Matrices - Untitled


Next: Explicit and Implicit Conversions,
Previous: Creating Diagonal Matrices,
Up: Basic Usage


---

#### 20.1.2 Creating Permutation Matrices

For creating permutation matrices, Octave does not introduce a new function, but
rather overrides an existing syntax: permutation matrices can be conveniently
created by indexing an identity matrix by permutation vectors.
That is, if q is a permutation vector of length n, the expression

```
       P = eye (n) (:, q);
```

will create a permutation matrix - a special matrix object.

```
     eye (n) (q, :)
```

will also work (and create a row permutation matrix), as well as

```
     eye (n) (q1, q2).
```

For example:

```
       eye (4) ([1,3,2,4],:)
     =>
     Permutation Matrix
     
        1   0   0   0
        0   0   1   0
        0   1   0   0
        0   0   0   1
     
       eye (4) (:,[1,3,2,4])
     =>
     Permutation Matrix
     
        1   0   0   0
        0   0   1   0
        0   1   0   0
        0   0   0   1
```

Mathematically, an identity matrix is both diagonal and permutation matrix.
In Octave, `eye (n)` returns a diagonal matrix, because a matrix
can only have one class. You can convert this diagonal matrix to a permutation
matrix by indexing it by an identity permutation, as shown below.
This is a special property of the identity matrix; indexing other diagonal
matrices generally produces a full matrix.

```
       eye (3)
     =>
     Diagonal Matrix
     
        1   0   0
        0   1   0
        0   0   1
     
       eye(3)(1:3,:)
     =>
     Permutation Matrix
     
        1   0   0
        0   1   0
        0   0   1
```

Some other built-in functions can also return permutation matrices. Examples include
inv or lu.
